# Editorial: Innovations in depression diagnosis and treatment outcome monitoring

**DOI:** 10.3389/fdgth.2023.1224999

**Published:** 2023-06-09

**Authors:** Milena B. Čukić, Danka Savić, Maie Bachmann

**Affiliations:** ^1^ Empa Swiss Federal Labs for Materials Science and Technology, Biomimetic Membranes and Textiles, St. Gallen, Switzerland; ^2^ Vinča Institute of Nuclear Science, University of Belgrade, Belgrade, Serbia; ^3^ Biosignal Processing Laboratory, Tallinn University of Technology, Tallinn, Estonia

**Keywords:** depression, electrophysiology, data-based computational psychiatry, remote patient monitoring, agent-based modeling (ABM)

**Editorial on the Research Topic** Innovations in depression diagnosis and treatment outcome monitoring

After WHO already made the prognosis in 2017 that depression would become the first world-wide reason of work disability by 2030, pandemic happened, causing economic changes and a sense of instability in many people’s realities. This demonstrably contributed to a surge in numbers of many psychiatric diagnoses and due to pandemic rules and restrictions we realized that mental health care increasingly became harder to access (1). This change in dynamics called out for innovative approaches to augment the effectivity of clinicians and to help patients to access the care they need. There are numerous contributions from technical sciences aimed at objective-based detection of biomarkers extracted from electrophysiological signals, under the name Data-based Computational Psychiatry. With higher acceptance of many Telehealth & IoT solutions, we would expect that already demonstrated methods of detection and possible remote monitoring of patients (RPM) would be introduced and translated to everyday clinical practice. On top of that, many AI applications already showed to be helpful in psychiatry. Once combined the collection of physiological data and self-reports (via mobile applications) with all kinds of portable devices, it can significantly improve the mood assessment, control of comorbidities and better understanding of real-time changes of the patients’ state. As telehealth &IoT application has shown in the COVID crisis, in many cases it is not necessary for patients to physically visit their dedicated specialists; the same could be done in psychiatry. This can also be used to augment other parts of health operations other than diagnostics and treatment. But contrary to many existent and validated telehealth solutions, we see none in psychiatry. Why is it so?

We obviously need to discuss and better understand the reasons for such low acceptance. We are aware of many problems like privacy and protection of the data issues, but electrophysiological recordings, for example are anonymized and GDPR compliant. There is numerous published research showing that the clinician can better inform their future decisions on diagnosis and treatment management of depression and increasingly recognize comorbidities easier, as well as prevent some adverse developments. For example, a frequent comorbidity to depression is a cardiovascular disease that could be screened early by a portable device and consequent nonlinear HRV analysis, hence the therapy could be adapted accordingly. HRV indices can also be informative about the phase of disease (or progress of the therapy), the severity of disease but also about the effectivity of the therapy, which can become increasingly important in everyday clinical practice. We recently demonstrated not only that nonlinear analysis of CVD risks in depression is having much greater effect size in detection (2), but in more general sense, that nonlinear analysis is more reliable even in detection from seizmocardiogram (SCG) and gyrocardiogram (GCG) (that could be potentially used interchangeably to ECG) (3).

Some researchers showed that EEG analysis can help improve diagnostics, but also detect the responders to certain kinds of therapy (such as ECT, rTMS, or tDCS) especially with application of nonlinear signal analytics (4). Cardio-vagal control is well understood and explored (corroborated by an impressive quantity of publications) that it is fascinating that clinicians are not using it in their practice to take better care of their patients. And save the time so they can help more people on waiting lists. Part of this technical development is due to enormous rise of various AI methods, that are already in use in many other areas, but in medicine the average acceptance time for any innovation is 17 years, as it was to routinely washing hands before the exam of the patient. It is understandable that any innovation in medicine should be rigorously examined before accepted, but in electrophysiology, which is basically the oldest among imaging methods (and the low-cost non-invasive methodology), this would be just a re-use of already meticulously examined practices adding just another layer of analytics proven to be accurate.

This editorial will provide a brief overview of the innovative approaches proposed for use in psychiatry described and discussed in the four studies featured in this Research Topic, in order to demonstrate that the solutions from technical sciences/engineering are at grasp, but need to be discovered, translated and incorporated in wider clinical practice. This Research Topic attracted the work of several important issues addressed in this regard.

First, a state-of-the art detection of depression and possible monitoring of progress in outpatients’ population (Zitouni et al.) In this particular paper a computational intelligence tool for the automated detection of MDD with and without suicidal ideation is presented. Zitouni et al. managed to automatically identify the disorder severity in MDD patients using multi-modal physiological signals recordings, including electrocardiogram (ECG), finger photoplethysmography (PPG) and respiratory signals (RSP). The authors first performed a literature review, although the majority of considered papers on depression detection were based on electroencephalogram (EEG), whilst this research opted for ECG and respiration, that are in our opinion even the stronger candidates for this type of detection due to cortico-vagal control and its proven abnormality in mood disorders. In their work, the Authors extracted 11 nonlinear features and developed a methodology comprising of several layers of processing the raw signals in order to automate the classification (more than 2 classes) where SVM performed better than KNN. Although the validation shows that this approach to classification is promising, the essential value of this work is that the detection of existent suicide risk could more likely be early detected if both ECG and respiratory signals were used (instead of EEG), since the physiological link is more precisely targeting this disbalance in severe depression. Although the authors did not include closer physiological interpretation (they focused on determining the most relatable features that led to accurate classification), the real question is, in this particular area of research, when (and if) those promising results would be eventually translated to healthcare system. We can only guess whether that is the side effect of poor digitalization process in clinical psychiatry, or a very variable kinds of present equipment and very rare clinical use of wearables, or simple the fact that as in any innovation, the mainstream scientist does not like it, or majority of clinicians like their status quo. We hope that would change in near future, since we can see an escalation of waiting list especially in mental health care.

Another interesting approach to tackling mental health, as many others was inspired by pandemic crisis, when scientist tried to conclude, based on available databases and information about the workflow in clinical practice, how to organize that particular process better (Brice et al.). The authors modeled disease progression and treatment pathways in depression, relying on systems dynamics and agent-based modeling. This framework was able to quantify demand, service capacities (and costs) across all care pathways for a range of different scenarios. We can see that an improvement is possible, but decisions about that are made probably out of the clinical sector, and hence is lagging behind our present understanding of possible improvements.

In addition, in this bundle there are two papers that are addressing better navigation of mental health patients and offering solutions to that particular situation. One publication, of a pilot study (Weller et al.) showed that gamification can improve antidepression treatment, demonstrating that together with information elements this approach can significantly increase cognitive control training efficacy that leads to decrease of depressive symptoms. Yet another study (Frank et al.) showed that social rhythm principles based personalized digital intervention can significantly improve therapeutical outcomes in depression treatment.

To conclude, it is not that proposed solutions by variously oriented scientists are lacking, it is the very poor outreach and problematic procedure of acceptance (trust) and standard requirements for innovative techniques and technologies to really enter everyday clinical practice and increase its efficiency in terms of shorter times for patients to feel better, are lacking. Not many researchers are considering it essential, but in reality it is; in the current atmosphere when we are bombarded daily by the news on how automation and especially advanced use of artificial intelligence (and machine learning as its sub-field) can go rogue, not only regular citizens but also specialists and clinicians need additional reassurance in validity of developed models (especially trained on much larger datasets), and necessary regulatory framework to develop trust in applying safely what was developed in research.

We can only hope that in parallel to more detailed regulatory acts on AI application especially in medicine will also contribute to safe and secure use of part of it in medical applications and in particular in psychiatry. As reflected in these papers, there is a huge potential for collaborations between health professionals, data scientists, and engineers, and we hope this Research Topic is a step in that direction.
